# Baf60c in skeletal muscle regulates adipose tissue thermogenesis via Musclin-mediated endocrine signaling

**DOI:** 10.1093/lifemeta/loaf015

**Published:** 2025-05-04

**Authors:** Shuang Han, Lu Jin, Wei Peng, Xue Lv, Ziyin Zhang, Tongyu Liu, Lin Mi, Yue Gao, Jun-fen Fu, Zhuo-Xian Meng

**Affiliations:** Department of Geriatrics, Hangzhou First People’s Hospital, Hangzhou, Zhejiang 310006, China; Department of Endocrinology, Children’s Hospital, Zhejiang University School of Medicine, National Clinical Research Center for Child Health, Hangzhou, Zhejiang 310052, China; Department of Endocrinology, Children’s Hospital, Zhejiang University School of Medicine, National Clinical Research Center for Child Health, Hangzhou, Zhejiang 310052, China; Department of Geriatrics, Hangzhou First People’s Hospital, Hangzhou, Zhejiang 310006, China; Department of Pathology and Pathophysiology and Department of Cardiology of the Second Affiliated Hospital, Zhejiang University School of Medicine, Hangzhou, Zhejiang 310058, China; Department of Geriatrics, Hangzhou First People’s Hospital, Hangzhou, Zhejiang 310006, China; Department of Pathology and Pathophysiology and Department of Cardiology of the Second Affiliated Hospital, Zhejiang University School of Medicine, Hangzhou, Zhejiang 310058, China; Department of Cell and Developmental Biology, Life Sciences Institute, University of Michigan, Ann Arbor, MI 48109, United States; Department of Cell and Developmental Biology, Life Sciences Institute, University of Michigan, Ann Arbor, MI 48109, United States; Department of Geriatrics, Hangzhou First People’s Hospital, Hangzhou, Zhejiang 310006, China; Department of Endocrinology, Children’s Hospital, Zhejiang University School of Medicine, National Clinical Research Center for Child Health, Hangzhou, Zhejiang 310052, China; Department of Geriatrics, Hangzhou First People’s Hospital, Hangzhou, Zhejiang 310006, China; Department of Pathology and Pathophysiology and Department of Cardiology of the Second Affiliated Hospital, Zhejiang University School of Medicine, Hangzhou, Zhejiang 310058, China

**Keywords:** Baf60c, Musclin, Mef2c, chromatin remodeling, thermogenic metabolism

## Abstract

Skeletal muscle plays a key role in metabolic homeostasis. Brg1/Brm-associated factor (Baf) 60c, a subunit of the mating type switching/sucrose non-fermenting (SWI/SNF) chromatin remodeling complexes, was previously identified to be robustly involved in glycolytic muscle function and systemic metabolic balance. However, whether Baf60c regulates the secreted factors and couples the skeletal muscle function to systemic metabolism remains unclear. Here, we uncover that Baf60c regulates the expression of a series of secreted factors, among which Musclin, a recently identified negative regulator of beige adipocyte thermogenesis, was top-ranked in the upregulated factors in *Baf60c*-deficient muscle. Mechanistically, Baf60c physically interacts with the transcription factor myocyte enhancer factor 2c (Mef2c) and modulates the chromatin accessibility at the proximal promoter regions upstream of the *Musclin* gene transcription start site (TSS), therefore negatively regulating *Musclin* gene expression in the skeletal muscle. Further *in vivo* metabolic assays demonstrate that muscle-specific *Baf60c* ablation inhibits thermogenesis and elevates blood glucose. Conversely, muscle-specific overexpression of *Baf60c* increases thermogenesis and energy expenditure and improves systemic glucose metabolism. Together, this work uncovers Baf60c/Mef2c-mediated chromatin remodeling signaling in myocytes that control adipose tissue thermogenesis and systemic metabolism through Musclin-mediated muscle-fat crosstalk.

## Introduction

Skeletal muscle works not only as an exercise organ but also as an important secretory organ in our human body. It has been uncovered that muscle-secreted factors, which are also regarded as myokines, actively participate in maintaining systemic homeostasis by mediating crosstalk among tissues [1, 2]. To date, more than 600 myokines have been identified by proteomic approaches in the skeletal muscle [3]. Recent work has demonstrated that most myokines take part in nutrient sensing and coordinate metabolic homeostasis [1, 4–8]. However, how they are regulated in response to environmental changes, including nutrition, temperature, infection, etc., remains largely unknown and is also gaining increasing attention in translational medicine.

Brg1/Brm-associated factor (Baf) 60c is a key component of the mating type switching/sucrose non-fermenting (SWI/SNF) chromatin-remodeling complex which could directly interact with transcription factors (TFs) [9]. The Baf60c protein is localized primarily in the cell nucleus and is expressed in a variety of tissues. A previous study indicated that Baf60c regulates muscle differentiation-related gene expression through interaction with myogenic differentiation 1 (MyoD) [10]. Our studies found that Baf60c regulates glucose metabolism through the Baf60c-DEP domain-containing mTOR-interacting protein (Deptor)-Akt signaling axis in skeletal myofibers [11], and works as a key factor participating in skeletal muscle regeneration through regulating the expression of a myokine dickkopf3 (DKK3) and its mediated paracrine action [4]. However, whether Baf60c is actively involved in the systemic metabolic regulation coordinating with other tissues through chromatin remodeling effect in the expression of secreted factor-associated genes remains further investigated.

In this study, it is quite interesting to reveal that Baf60c in the skeletal muscle negatively regulates the expression of Musclin, a recently reported myokine involved as an inducible negative regulator in beige adipocyte thermogenesis via the endocrine mechanism [1]. Chromatin accessibility profiling assay revealing the increased “openness” in the proximal regions upstream of the *Musclin* gene transcription start site (TSS) upon Baf60c inactivation indicates that Baf60c may affect the *Musclin* gene transcription. Motif analysis and the following co-immunoprecipitation (Co-IP) and gene expression regulation assays indicated that Baf60c may recruit the TF myocyte enhancer factor (Mef) 2c, directly regulating *Musclin* gene expression in a cell-autonomous manner. Further *in vivo* metabolic assays demonstrate that Baf60c in the skeletal muscle can profoundly influence thermogenesis and systemic glucose metabolism and energy expenditure. Therefore, this work uncovers that Baf60c cell autonomously regulates Musclin expression through recruiting TF Mef2c in myofibers and may work as a key player in thermogenic metabolism via the autocrine mechanism.

## Results

### Baf60c negatively regulates Musclin expression in skeletal muscle

**Figure 1:**
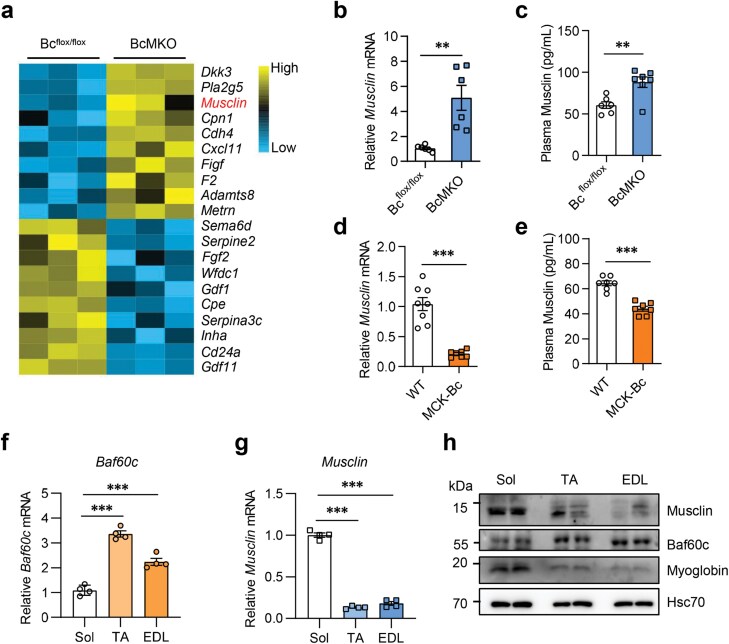
Baf60c negatively controls Musclin expression in skeletal muscle. (a) Heatmap depicting the expression of the top 10 upregulated and the top 10 downregulated muscle secreted protein-encoding genes ranked by fold-change (FC) in quadriceps muscles of 3-month-old BcMKO mice (cutoff: *P* < 0.05 and log_2_(FC) > 0.5) compared to the control. Bc^flox/flox^, control mice carry two loxp sites flanked in exon 2 of the mouse *Baf60c* gene; BcMKO, muscle-specific *Baf60c* knockout mice. (b and c) *Musclin* gene expression in skeletal muscles (*n* = 6 per group) (b) and Musclin secretion levels in plasma (*n* = 6−7 per group) (c) of BcMKO and control mice. (d and e) *Musclin* expression in skeletal muscles (*n* = 7−8 per group) (d) and Musclin secretion levels in plasma (*n* = 7 per group) (e) from MCK-Bc and control mice. WT, wildtype control mice; MCK-Bc, muscle creatine kinase promoter-driven *Baf60c* transgenic mice. (f and g) *Baf60c* (f) and *Musclin* (g) mRNA levels in different muscles of WT mice (*n* = 4 per group). ^***^P < 0.001, by one-way ANOVA with multiple comparisons. Sol, soleus; TA, tibialis anterior; EDL, extensor digitorum longus. (h) Musclin protein levels in different muscles of WT mice. Data represent mean ± SEM. ^**^P < 0.01; ^***^P < 0.001, by two-tailed unpaired Student’s *t*-test, unless otherwise noted.

Previous studies found that Baf60c is a specific chromatin remodeling component for gene transcription in metabolic organs [9, 12, 13]. This may not only regulate the metabolism locally but also affect systemic metabolic homeostasis through secreted factor-mediated organ−organ crosstalk. To explore whether Baf60c regulates the transcription of secreted factors in the skeletal muscle that link to systemic metabolism, we performed microarray detection using quadriceps muscles from 3-month-old muscle-specific *Baf60c* knockout (BcMKO) and control mice (Bc^flox/flox^) with normal housing and feeding conditions and without any additional treatment. A series of secreted factors were differentially expressed upon *Baf60c* knockout with the transcription levels of top-ranked differentially expressed secreted factors shown in the heatmap (Fig. 1a). Interestingly, among those genes, a nutrition-responsive muscle-secreted factor, Musclin, is dramatically up-regulated in BcMKO mice compared with the control group (Fig. 1a). Musclin is also identified to be an important regulator in adipocyte thermogenesis via the endocrine mechanism [1, 14]. To confirm the regulation of *Musclin* expression by Baf60c, we examined the *Musclin* mRNA level in skeletal muscle and the Musclin protein level in plasma from BcMKO mice. As expected, compared to the control mice, both the mRNA level and plasma concentration of Musclin were robustly increased in BcMKO mice (Fig. 1b and c). Conversely, the mRNA level and plasma Musclin were both significantly decreased in muscle-specific Baf60c transgenic (MCK-Bc) mice (Fig. 1d and e). Consistent with our previous observation [15], the expression of Baf60c was lower in soleus (Sol), a representative of oxidative muscles, than that of tibialis anterior (TA) and extensor digitorum longus (EDL), muscles with more glycolytic function (Fig. 1f and h). Intriguingly, the expression of Musclin at both the mRNA and protein levels was inversely proportional to that of Baf60c in Sol, TA, and EDL muscles (Fig. 1g and h). Together, these data demonstrate that Baf60c negatively regulates Musclin expression in the skeletal muscle.

### Baf60c regulates *Musclin* expression in a cell-autonomous manner

**Figure 2:**
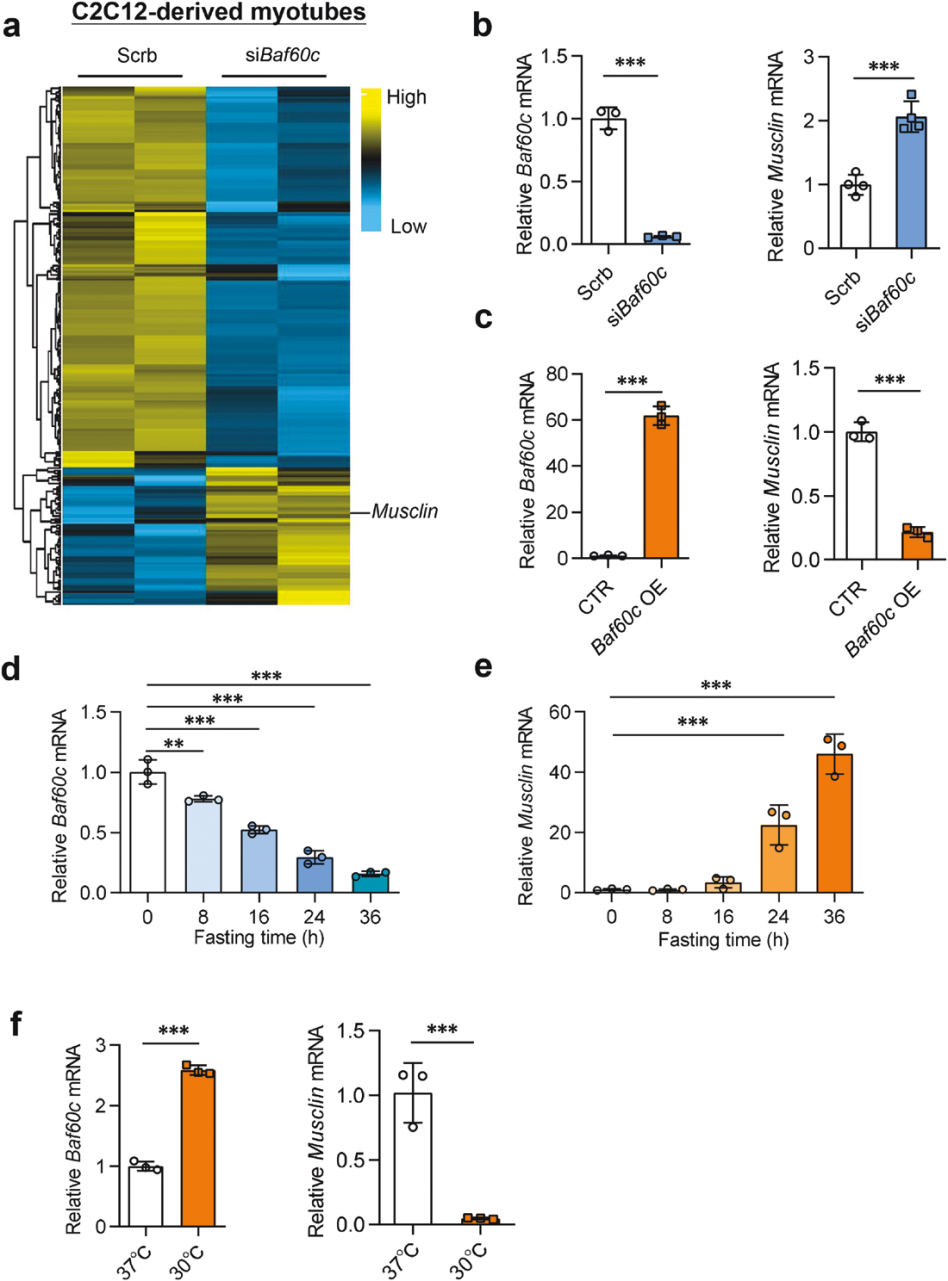
Baf60c regulates *Musclin* expression in a cell-autonomous manner. (a) Heatmap depicting the expression of upregulated and downregulated genes in C2C12-derived myotubes with *Baf60c* knockdown (cutoff: *P* < 0.05 and |average change| > 1) compared to the control myotubes. (b) The *Baf60c* (left) and *Musclin* (right) mRNA expression in *Baf60c* siRNA-mediated knockdown and scramble siRNA-treated control myotubes (*n* = 3 per group). (c) The *Baf60c* (left) and *Musclin* (right) mRNA expression in *Baf60c* OE C2C12 cells (*n* = 3 per group). (d and e) The *Baf60c* (d) and *Musclin* (e) mRNA expression in C2C12-differentiated myotubes after starvation for 0, 8, 16, 24, and 36 h (*n* = 3 per group). During starvation, cells were treated by DMEM supplemented with low glucose (1 mmol/L) and 0.1% BSA. ^***^P < 0.001, by one-way ANOVA with multiple comparisons. (f) The *Baf60c* (left) and *Musclin* (right) mRNA expression in myotubes cultured at different temperatures (*n* = 3 per group). Data represent mean ± SD. ^***^P < 0.001, by two-tailed unpaired Student’s *t*-test, unless otherwise noted.

To verify whether the *Musclin* expression is cell-autonomously regulated, we re-analyzed microarray data (GSE79925) performed previously from C2C12-derived myofibers with *Baf60c* siRNA-mediated knockdown and scramble siRNA-treated control cells. The result shows that *Musclin* expression is also among those mRNA level-increased genes in the si*Baf60c*-treated group (Fig. 2a). The relative *Baf60c* and *Musclin* mRNA levels were further confirmed by quantitative PCR (qPCR) analysis (Fig. 2b). We next overexpressed *Baf60c* in C2C12 cells, and detected *Musclin* mRNA levels after the cells were fully differentiated. As expected, the relative *Musclin* mRNA levels were sharply reduced in *Baf60c* overexpressed (*Baf60c* OE) cells compared to the control group (Fig. 2c). To further investigate the regulation of *Musclin* expression by Baf60c in myofibers, we measured the mRNA levels of both *Baf60c* and *Musclin* in differentiated C2C12 cells after glucose starvation for different times. As Baf60c is reported to be a key factor in sensing glucose in myofibers [16], it continuously decreased in mRNA level during glucose starvation (Fig. 2d), while, notably, the mRNA content of *Musclin* was increasingly elevated over time (Fig. 2e). Interestingly, low temperature (30°C) induced the increased expression of *Baf60c* and the decreased expression of *Musclin* in differentiated C2C12 cells (Fig. 2f). These data suggest that Baf60c regulates *Musclin* expression in a cell-autonomous manner.

### Baf60c regulates *Musclin* transcription in coordination with Mef2c in skeletal muscle

**Figure 3:**
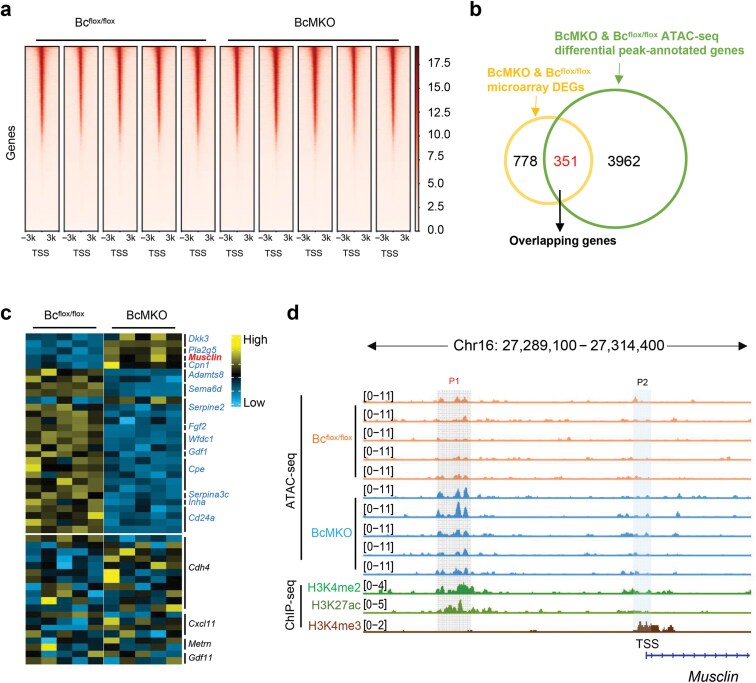
Baf60c regulates *Musclin* transcription through modification of chromatin accessibility. (a) Chromatin accessibility analysis in quadriceps muscles from 3-month-old BcMKO and Bc^flox/flox^ mice based on ATAC-seq data. (b) Venn diagram of DEGs from microarray data of BcMKO and Bc^flox/flox^ mice mentioned in Fig. 1a (cutoff: *P* < 0.05 and log_2_(FC) > 0.5) and differential peak-annotated genes in ATAC-seq data indicated in Fig. 3a (cutoff: *P*_adjust_ < 0.05 and log_2_(FC) > 0.5). (c) Heatmap depicting the differential peaks in ATAC-seq analysis associated with secreted proteins mentioned in Fig. 1a. (d) Genome browser tracks of ATAC-seq of BcMKO and Bc^flox/flox^ mice and the indicated ChIP-seq data in the *Musclin* gene locus. H3K4me3 and H3K27ac are well-known histone hallmarks for transcription initiation and active enhancers, respectively, and H3K4me2 is reported to modulate the stability of RNA polymerase Ⅱ pausing.

Baf60c, as a transcriptional cofactor, alters the local chromatin structure and orchestrates the transcriptional responses through mediating the recruitment of the SWI/SNF complexes and TFs to selective loci [15]. Transposase-accessible chromatin with high-throughput sequencing (ATAC-seq) was performed on nuclei isolated from quadriceps to profile the chromatin accessibility difference between 3-month-old BcMKO and control mice (Fig. 3a). For sequencing quality assessment, we evaluated the DNA fragment size distribution in the established DNA library before sequencing (Supplementary Fig. S1). More than 4300 genes with differential peaks in their proximal regions of their TSS in BcMKO mice compared to the control group were identified through ATAC-seq. Among these genes, 351 ones were differentially expressed at the mRNA level based on the overlapping analysis of BcMKO microarray differentially expressed genes (DEGs) and BcMKO ATAC-seq differential annotated genes (Fig. 3b), indicating that Baf60c may be actively involved in the transcription regulation of those overlapped genes (Fig. 3b). Furthermore, part of the secreted factor-encoding genes shown in Fig. 1a were observed with differential peak(s) in the proximal region of their TSS, among which *Musclin* showed an increased peak in BcMKO mice (Fig. 3c). The increased chromatin accessibility in the proximal upstream of *Musclin* TSS between BcMKO and control mice was better visualized from the Integrative Genomics Viewer (IGV) plot (Fig. 3d), in which histone modification signals of di-methylated histone H3 lysine 4 (H3K4me2), tri-methylated histone H3 lysine 4 (H3K4me3), and acetylated histone H3 lysine 27 (H3K27ac) at the same loci are also shown according to the indicated chromatin immunoprecipitation followed by high-throughput sequencing (ChIP-seq) data. H3K4me3 and H3K27ac are well-known as a histone hallmark for transcription initiation [17] and a marker for active enhancers [18], respectively, and H3K4me2 is reported to modulate the stability of RNA polymerase Ⅱ pausing [19]. The higher peak annotated to *Musclin* in BcMKO mice was perfectly localized in the region of robust H3K27ac and H3K4me2 modification signal, though no difference of chromatin accessibility was observed around the *Musclin* TSS region marked by H3K4me3 modification (Fig. 3d). These data imply that Baf60c may regulate *Musclin* transcription through modification of chromatin accessibility in the proximal region of the *Musclin* gene locus.

**Figure 4:**
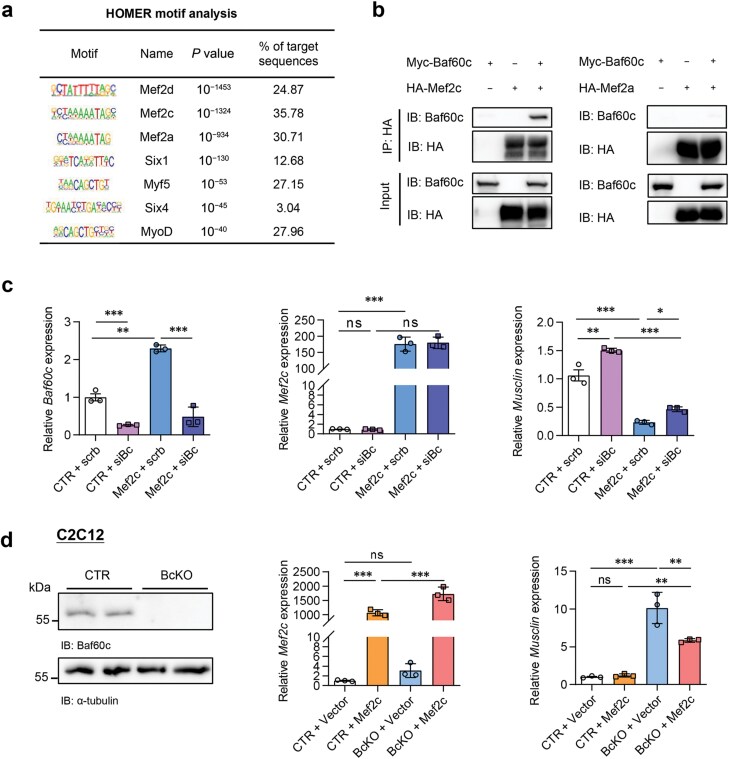
Baf60c regulates Musclin expression through interacting with Mef2c. (a) HOMER motif analysis on ATAC-seq data of quadriceps muscles from 3-month-old Bc^flox/flox^ and BcMKO mice. (b) Physical interaction of Baf60c with Mef2a and Mef2c in HEK293T cells. HEK293T cells were transiently transfected with Myc-tagged Baf60c (Baf60c) and Flag/HA-tagged Mef2a or Mef2c, followed by IP with anti-HA agarose beads and immunoblotting. (c) qPCR analysis of the *Baf60c* (left), *Mef2c* (middle), and *Musclin* (right) gene expression in *Mef2c* overexpressed and control myotubes with either *Baf60c* knockdown or not (*n* = 3). (d) The Baf60c expression in *Baf60c* knockout C2C12 cells (left panel), and qPCR analysis of the *Mef2c* (middle panel) and *Musclin* (right panel) gene expression in *Baf60c* knockout and control myotubes with either *Mef2c* overexpression or not (*n* = 3). Data represent mean ± SD. ^*^P < 0.05; ^**^P < 0.01; ^***^P < 0.001; ns, no significance, by one-way ANOVA with multiple comparisons.

As Baf60 is reported to play important roles in transcription regulation through recruiting TFs and linking them to SWI/SNF chromatin remodeling complex [20], we then ask whether Baf60c is directly involved in *Musclin* transcription and which is the key TF. We first performed known motif enrichment analysis on the differential accessible peaks (DAPs) according to ATAC-seq data of skeletal muscles from BcMKO and control mice, and multiple TFs were significantly enriched. The top-ranked and muscle tissue-enriched TFs are shown in Fig. 4a, including Mef2d, Mef2c, Mef2a, sine oculis homeobox homolog 1 (Six1), myogenic factor 5 (Myf5), Six4, and MyoD. We then visualized the binding signals of Mef2d, Six1, Myf5, Six4, and MyoD in the proximal region of *Musclin* gene locus via IGV based on previously published ChIP-seq data. However, no binding signals of the five TFs were detected in either H3K4me3-marked transcription initiation region or H3K27ac-marked active enhancer region (Supplementary Fig. S2), which led us to detect the role of Mef2a and Mef2c in the regulation of *Musclin* gene expression. Co-IP assay performed in HEK293T cells verified the direct physical interaction of Baf60c with Mef2c, but not with Mef2a (Fig. 4b). We further analyzed the gene expression in *Mef2c* stably overexpressed C2C12-differentiated myofibers upon adenovirus-mediated knockdown of *Baf60c*. As expected, the cells were successfully expressed with *Mef2c* upregulation and *Baf60c* downregulation. Interestingly, *Mef2c* overexpression significantly inhibited *Musclin* expression, which was partially reversed by *Baf60c* knockdown (Fig. 4c). Similarly, *Baf60c* knockout increased *Musclin* expression, which was inhibited by *Mef2c* overexpression (Fig. 4d). The expression of *Musclin* was also measured following *Mef2c* knockdown mediated by siRNA transfection. As expected, *Musclin* level was significantly increased in *Mef2c*-knockdown C2C12 myotubes (Supplementary Fig. S3a). To confirm the regulatory pattern *in vivo*, we tested the interaction between Baf60c and Mef2c by performing endogenous IP using quadriceps muscle samples from WT and muscle-specific Flag-HA tagged *Baf60c* transgenic mice (MCK-Bc). The results confirmed that endogenous Mef2c interacted with Baf60c in MCK-Bc muscle *in vivo* (Supplementary Fig. S3b). To further investigate whether Baf60c and Mef2c are recruited to the same promoter regions of the *Musclin* gene to regulate its transcription, we performed a ChIP-qPCR assay with antibodies against endogenous Baf60c and Mef2c. The results showed that both Baf60c and Mef2c were recruited to the proximal *Musclin* promoter region (Supplementary Fig. S3c). This finding is consistent with the open region near the TSS in muscle from BcMKO mice compared to the controls, as revealed by ATAC-seq (Fig. 3d). Those data indicate that Mef2c might be the key transcription factor recruited by Baf60c in the regulation of *Musclin* expression in myofibers.

### Baf60c deficiency in skeletal muscle inhibits thermogenesis and systemic glucose metabolism in response to cold temperature

**Figure 5:**
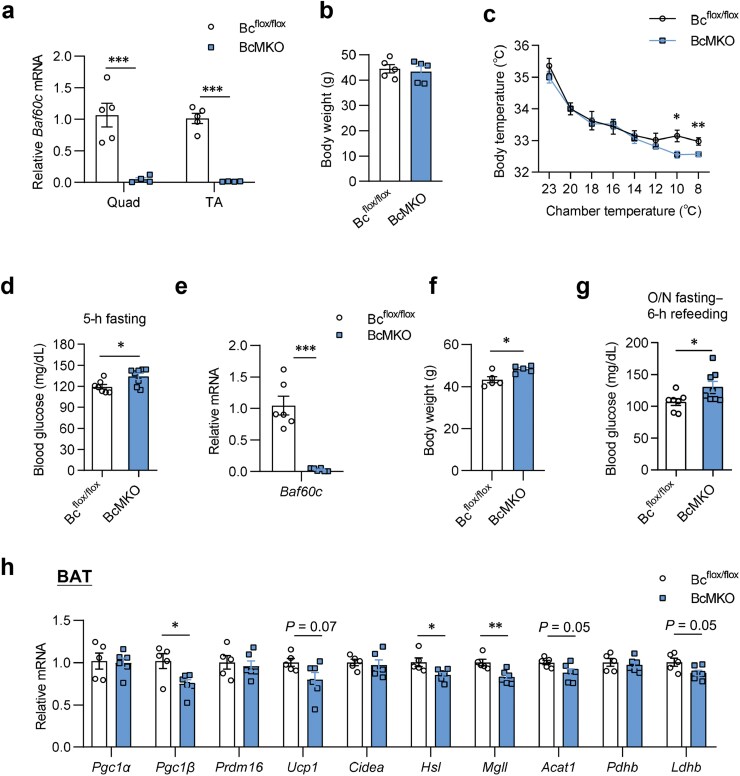
Metabolic regulation in BcMKO mice. (a−c) The phenotypes of BcMKO and control mice under chow diet feeding. (a) *Baf60c* expression in Quad and TA muscles (*n* = 4−5 per group). Quad, quadriceps; TA, tibialis anterior. (b) Body weight under chow diet feeding (*n* = 5 per group). (c) Core body temperature during cold acclimation (*n* = 7−8 per group). ^*^P < 0.05; ^**^P < 0.01, by two-way ANOVA with multiple comparisons. (d) The 5-h fasting blood glucose levels at 6°C during cold acclimation (*n* = 7−8 per group). (e−h) The phenotypes of BcMKO and control mice under HFD feeding housed in a 16°C chamber. (e) The gene expression in Quad muscles (*n* = 6 per group). (f) Body weight after HFD feeding in chronic cold temperature (*n* = 5 per group). (g) Blood glucose levels after 6-h refeeding following overnight (O/N) fasting in mice (*n *= 7−8 per group). (h) Gene expression in brown adipose tissue (*n* = 5−6 per group). BAT, brown adipose tissue. Data represent mean ± SEM. ^*^P < 0.05; ^**^P < 0.01; ^***^P < 0.001, by two-tailed unpaired Student’s *t*-test, unless otherwise noted.

We previously reported that Baf60c in the skeletal muscle functions as a glucose sensor and plays a key role in systemic energy homeostasis [11]. Additionally, we have shown that Musclin is crucial for thermogenic metabolism and the development of obesity. Specifically, elevated Musclin expression suppresses thermogenic activity in adipocytes and exacerbates diet-induced obesity, while Musclin blockade, through genetic ablation or a neutralizing antibody, promotes beige fat thermogenesis and improves systemic metabolic homeostasis [1]. Building on these findings, we further investigated the role of Baf60c in thermogenic metabolism, as Baf60c negatively regulates *Musclin* expression in skeletal muscle (Fig. 1). To test this, BcMKO and control mice (Fig. 5a) in chow diet feeding, with no significant body weight difference (Fig. 5b), were firstly subjected to acute cold exposure. Interestingly, BcMKO mice displayed impaired thermogenesis and were more likely unable to maintain body temperature compared to the control mice (Fig. 5c), as well as exhibiting inhibited glucose regulation during cold acclimation (Fig. 5d). We continued to investigate the metabolic phenotypes between the BcMKO and control mice upon high-fat diet (HFD) feeding housed in chronic cold (16°C) condition. *Musclin* knockdown was also validated in quadriceps after HFD treatment in cold temperature (Fig. 5e). After HFD feeding in chronic cold temperature for 3 months, the body weight of BcMKO mice appeared to be significantly higher than the control mice (Fig. 5f). Moreover, the blood glucose levels were higher in  BcMKO mice after 6-h refeeding following overnight fasting (Fig. 5g). From the molecular aspect, we observed that thermogenesis- and lipolysis-associated genes were down-regulated due to the deficiency of *Baf60c* in muscle tissues (Fig. 5h). These results suggested that the decreased Baf60c expression in skeletal muscle may inhibit thermogenesis and glucose metabolism in response to cold temperature.

### Baf60c gain-of-function in skeletal muscle promotes thermogenic metabolism

**Figure 6:**
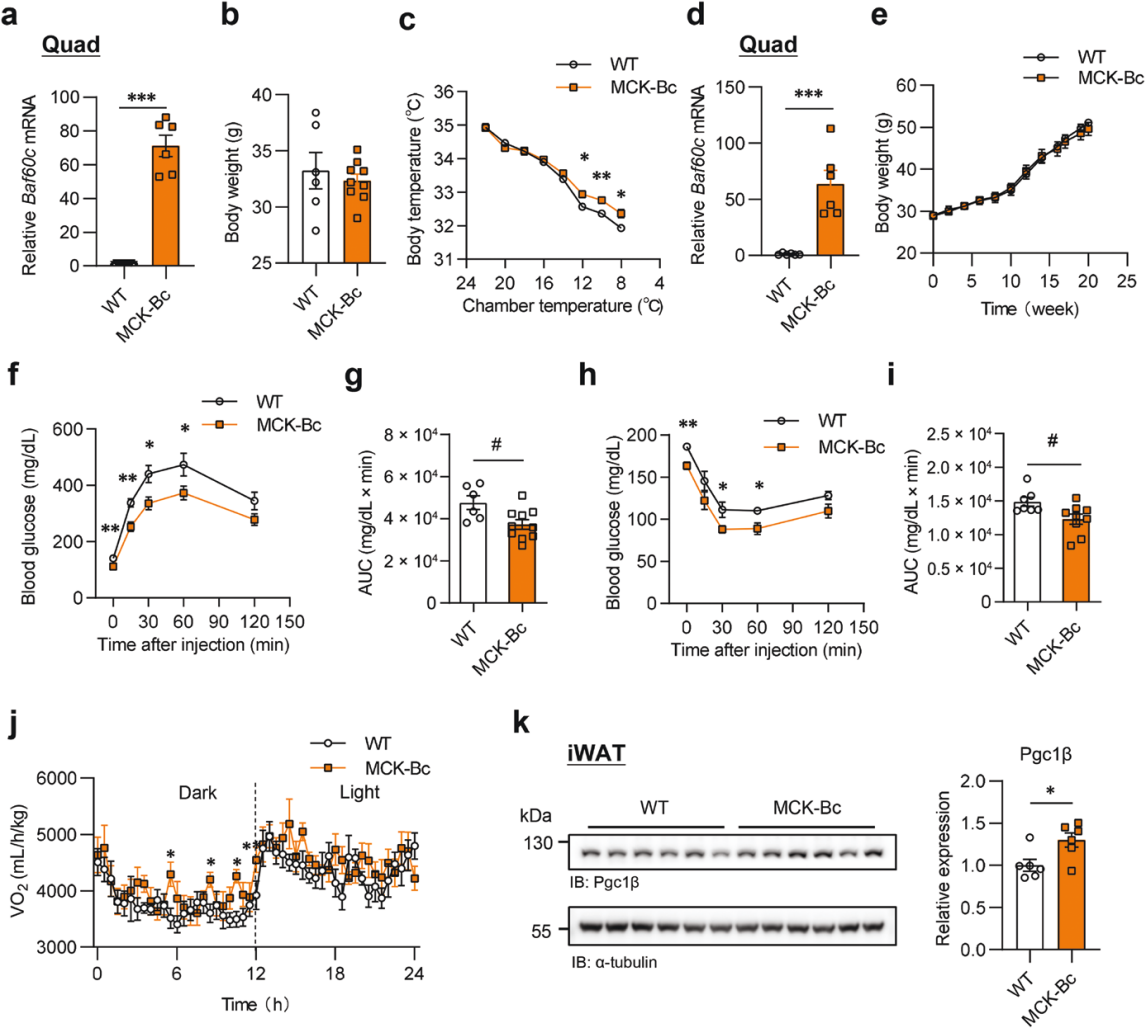
Metabolic regulation in muscle-specific *Baf60c* overexpression mice. (a−c) The phenotypes of MCK-Bc and control mice under chow diet feeding. (a) *Baf60c* gene expression in Quad. (b) Body weight under chow diet feeding at the age of 6 months (*n* = 6−9 per group). (c) Core body temperature during cold acclimation (*n* = 5−6 per group). ^*^P < 0.05; ^**^P < 0.01, by two-way ANOVA with multiple comparisons. (d−k) The phenotypes of MCK-Bc and control mice under HFD feeding. (d) Gene expression in Quad. (e) Body weight under HFD feeding in chronic cold condition. (f and g) GTT (f) and the area under curve (AUC) (g) (*n* = 6−10 per group). (h and i) ITT (h) and the AUC (i). Data represent mean ± SEM (*n* = 7−9 per group). (j) The O_2_ consumption rate of indicated mice monitored at 30°C. Mice were fed with HFD for 3 months. (k) The Pgc1β protein levels in iWAT (left panel) and the quantification (right panel). iWAT, inguinal white adipose tissue. Data represent mean ± SEM. ^*^P < 0.05; ^**^P < 0.01; ^***^P < 0.001; ^#^*P* < 0.05, by two-tailed unpaired Student’s *t*-test, unless otherwise noted.

Conversely, to test whether *Baf60c* overexpression in skeletal muscle promotes whole-body thermogenesis and energy expenditure, we first established MCK-Bc mice (Fig. 6a). Compared with the control mice, the MCK-Bc mice had no body weight difference under chow diet feeding (Fig. 6b). Similarly, we subjected MCK-Bc and control mice to cold acclimation. Interestingly, the MCK-Bc mice maintained the body temperature better during cold exposure compared to the control mice (Fig. 6c). Upon HFD feeding in 16°C chronic cold condition, *Baf60c* gene expression preserved much higher level in the MCK-Bc mice than that of the control mice (Fig. 6d). Though body weight showed no difference between these two groups (Fig. 6e), the glucose tolerance test (GTT) and insulin tolerance test (ITT) demonstrated that elevation of Baf60c in skeletal muscle much improved both glucose tolerance and insulin sensitivity (Fig. 6f−i). In addition, the whole metabolism of mice upon HFD feeding in chronic cold temperature was monitored using PhenoMaster TSE systems. Both the oxygen consumption rate and energy expenditure of the MCK-Bc mice exhibited to be higher than that of the control mice at dark (Fig. 6j; Supplementary Fig. S4a and b), while no differences in food intake and water consumption were observed (Supplementary Fig. S4c and d). Intriguingly, a key mitochondrial function regulator peroxisome proliferator-activated receptor γ coactivator 1β (Pgc1β) was expressed at a significantly higher level in inguinal white adipose tissue (iWAT) from HFD-fed MCK-Bc mice housed in 16°C, which is right opposite to that of MCK-Musclin mice (Fig. 6k). These results indicate that *Baf60c* overexpression in skeletal muscle promotes thermogenic metabolism during cold temperatures. However, interestingly, those metabolic benefits resulting from Baf60c during chronic cold exposure were reversed upon Musclin upregulation in circulation mediated by adeno-associated virus (AAV)-Musclin transduction (Supplementary Fig. S5). Firstly, the data showed that AAV-Musclin treatment led to a significantly higher plasma Musclin level in the plasma in the MCK-Bc mice (Supplementary Fig. S5d). As expected, compared to the control mice, AAV-Musclin treatment significantly repressed the downregulation of blood glucose by Baf60c in the skeletal muscle (Supplementary Fig. S5b), while no influence on *Baf60c* gene expression was observed upon upregulation of circulating Musclin (Supplementary Fig. S5a). In the meantime, the increased body temperature in MCK-Bc mice was also decreased after AAV-mediated *Musclin* overexpression (Supplementary Fig. S5c). Taken together, these results suggest that *Baf60c* overexpression in the skeletal muscle boosts cold-induced thermogenic metabolism, which is partially associated with the inhibition of Musclin expression.

## Discussion

The crosstalk between the skeletal muscle and adipose tissues is critically linked to metabolic homeostasis under physiological conditions. It has been increasingly accepted that the myokines play important roles in maintaining physical health. Transcription factors have been identified to integrate the metabolic and stress cues into the transcriptional reprogramming of metabolic pathways [21–25]. In this study, we unveil that Baf60c, a component of the SWI/SNF chromatin remodeling complex, serves as an epigenetic rheostat of Musclin expression in the skeletal muscle and regulates thermogenesis and systemic energy homeostasis indirectly. Mechanistically, we identify the transcription factor Mef2c as the key downstream factor in mediating the transcriptional regulation of *Musclin* in the skeletal muscle by Baf60c.

The SWI/SNF chromatin-remodeling complex includes 11 subunits, among which the Baf60 subunits have been identified as a vital regulator in energy and glucose metabolism [18, 26, 27]. Baf60 is comprised of three subunits, Baf60a, Baf60b, and Baf60c, with distinct tissue distribution patterns. The subunits have emerged as critical linkers between the SWI/SNF complex and transcription factors to regulate target gene expression in different organs. Baf60c, encoded by the SWI/SNF actin binding chromatin remodeling complex D-3 (*Smarcd3*), is enriched in the skeletal muscle, accelerating muscle regeneration through regulating the expression of DKK3 [4]. Meanwhile, it has been reported to work as a glucose sensor in the skeletal muscle [11, 15, 28]. In this study, we also uncover the role of Baf60c in Musclin expression regulation and systemic thermogenesis and energy homeostasis by applying skeletal muscle-specific knockout and transgenic mice.

The TF Mef2c is a member of MADS-box family, and it has been identified as an important player in the early development of the skeletal muscle, immune cells, and endothelial cells [29]. Previous studies suggest that Mef2c regulates the myofiber organization and mediates skeletal muscle morphogenesis and differentiation [30, 31]. In this study, we demonstrate that Mef2c is recruited by Baf60c to be involved in the transcription inhibition of Musclin, a muscle-secreted factor downregulating adipose thermogenesis. Mef2a was also reported to act as an evolutionarily conserved transcriptional factor that regulates myogenesis [32]. However, our Co-IP assay displayed a weak binding affinity of Baf60c to Mef2a, but a strong interaction of Baf60c with Mef2c, suggesting that Mef2c might be involved more in the chromatin remodeling and transcription regulation by Baf60c.

This study reveals the key role of Baf60c in *Musclin* transcription regulation, which led us to suppose that Baf60c might significantly promote beige fat thermogenesis that is inhibited by Musclin reported in our previous study [1]. The results turned out to be that there was a modest difference in beige adipose thermogenesis between the mice with Baf60c manipulation in skeletal muscle and the control mice, though muscle Baf60c was verified to be involved in body temperature maintenance and glucose metabolism in response to cold temperature. These results may be related to the complex effects mediated by the multiple factors regulated by Baf60c, as indicated in Fig. 1a and Fig. 3c, which deserve further investigation.

In summary, our work identifies Baf60c as a key guide in regulating the expression of multiple secreted factors, including Musclin, a recently identified secreted factor negatively regulating beige adipocyte thermogenesis, through chromatin modeling. Baf60c may affect the chromatin accessibility in the proximal regions upstream of the *Musclin* gene TSS and recruit the transcription factor Mef2c, directly regulating *Musclin* gene expression in a cell-autonomous manner. In addition, *in vivo*, metabolic assays demonstrate that Baf60c in skeletal muscle is robustly involved in thermogenesis and the improvement of glucose metabolism in response to cold challenges.

### Limitations of the study

One limitation of this study was the absence of *Mef2c* conditional knockout mice, which could have been used to validate the Baf60c-Mef2c-Musclin regulatory pathway *in vivo*. In this study, we instead confirmed the regulatory relationship *in vivo* through endogenous IP using quadriceps samples from wildtype (WT) and MCK-Bc mice. Further investigation into the role of the Baf60c-Mef2c-Musclin pathway in muscle metabolism is also needed.

## Materials and methods

### Animal experiments

*Baf60c* flox/flox (Bc^flox/flox^) mice and *Baf60c* transgenic mice on the C57BL/6J background were generated as previously described [4]. Mice were housed in 12-h light/12-h dark cycle at an ambient temperature of 23°C, fed on a standard chow diet or HFD (D12492, Research Diets). All experiments were performed on male mice, and they were sacrificed using CO_2_. The tissues were then immediately frozen in liquid nitrogen. All animal studies were performed according to procedures approved by the University Committee on the Use and Care of Animals at Zhejiang University.

### AAV production and transduction

AAVs were purchased from ChuangRui Bio (Lianyungang, China), and the production and purification were performed as previously described [1]. Briefly, AAV293 cells were cultured in a humidified incubator containing 5% CO_2_ at 37°C, and were seeded on culture dishes and grown to appropriate 90% confluency before transfection. AAV shuttle vector carrying target gene, Delta F6 helper vector, and RC2/9 vector were transduced to cells using polyethylenimine (PEI; Polysciences, Cat# 23966-2). Cells were centrifuged at 1,000 rpm for 10 min at 72 h post-transfection to collect cell pellets. Then, the virus was isolated from pellets through a series of biochemical protocols. The viral titer was determined by qPCR assay with the standard curve generated by serial dilutions of the AAV shuttle vector. MCK-Bc and littermate control mice were injected with AAV-GFP or AAV-Musclin (approximately 5 × 10^11^ viral particles/mL in 100 μL/mouse) through tail vein injection.

### Cold exposure assay

For cold acclimation assays, mice were subjected to a temperature-controlled chamber, in which the temperature was decreased by two degrees every two days until reaching 6°C. The mice were maintained for 5 more days at 6°C. The core body temperature of the indicated mice was monitored using a portable intelligent digital thermometer (TH212).

### GTT and ITT

GTT and ITT were performed as described previously [1]. For GTT, mice were given glucose solution in saline through intraperitoneal injection (2 g/kg body weight) after fasting overnight (approximately 16 h). Subsequently, blood glucose levels were determined at indicated time points (0, 15, 30, 60, and 120 min after glucose injection). For ITT, mice were intraperitoneally injected with insulin solution in saline (1 unit/kg body weight) after fasting for 4 h. Then, blood glucose levels were measured at indicated time points (0, 15, 30, 60, and 120 min after insulin injection).

### Radioimmunoassay of plasma Musclin

The plasma Musclin was measured as described previously [1]. Briefly, the plasma was pretreated with aprotinin (500 KIU/mL), and measured with a radioimmunoassay kit (HY-076, Beijing Sino-UK Institute of Biological Technology, Beijing, China).

### Cell culture

HEK293T and C2C12 cells were cultured in Dulbecco’s modified Eagle’s medium (DMEM, 11995065, Gibco) supplemented with 10% fetal bovine serum (FBS, SE100-011, VisTech), streptomycin (50 μg/mL), and penicillin (50 U/mL, 15140122, Gibco). All cells were cultured and maintained at 37°C in a 5% CO_2_ incubator.

### Muscle cell differentiation

C2C12 myoblasts were purchased from the American Type Culture Collection (ATCC) and cultured in DMEM containing 10% FBS. After reaching more than 90% confluency, the culture medium was switched to DMEM containing 2% FBS. Myotubes were subjected to related treatment after full differentiation.

### Western blot and IP

Whole cells and tissues were lysed for protein extraction. Protein samples were separated by SDS-PAGE after quantified and subsequently transferred onto a polyvinylidene difluoride membrane (Millipore), followed by immunoblotting with primary antibodies listed below: anti-Baf60c (Invitrogen, PA5-41093, 1:500), anti-Musclin (Abcam, 1:1000), anti-Pgc1(α+β) (Abcam, ab72230, 1:1000), anti-α-tubulin (Sigma, T6199, 1:1000), anti-Hsc70 (Stressgen, SPA815, 1:1000), and anti-myogenin (Santa Cruz, sc-576, 1:1000). Secondary HRP-conjugated goat anti-mouse (A4416, Sigma; 1:5000) and goat anti-rabbit (A6154, Sigma; 1:5000) antibodies were used. For IP, a small portion of the HEK293T cell protein lysate was taken as input, and the remaining part was incubated with anti-HA Agarose (#26182, Thermo Fisher Scientific) on a rotator at 4°C overnight. For endogenic IP, the quadriceps tissues were grinded into a homogenate for protein lysate. A small portion of the protein lysate was taken as input, and the remaining part was incubated with anti-Flag Agarose (SA042001, Smart-Lifesciences) on a rotator at 4°C overnight. The IP samples were washed with IP wash buffer five times and boiled with 1 × loading buffer for immunoblotting analysis. IP and input samples were separated by SDS-PAGE after quantified and subsequently transferred onto a polyvinylidene difluoride membrane (Millipore), followed by immunoblotting with primary antibodies listed below: anti-Baf60c (Invitrogen, PA5-41093, 1:500), anti-HA (Abclonal, AE036, 1:1000), anti-Flag (Proteintech, 20543-1-AP, 1:1000), anti-α-tubulin (Sigma, T6199, 1:1000), anti-Mef2c (Abcam, ab211493, 1:1000), and anti-Baf47 (Bethyl laboratories, A301-087A, 1:1000).

### RNA extraction and qPCR analysis

A commercial kit (DP430, TIANGEN Biotech) was used for total RNA extraction from adipose tissues. TRIzol (Invitrogen) reagent was used for RNA extraction from cells and muscle tissues. RNA reverse transcription was performed using the High-Capacity cDNA Reverse Transcription Kit (R222-01, Vazyme), and cDNA was analyzed by real-time qPCR through the Applied Biosystems (Thermo Fisher Scientific, USA). Relative gene expression was measured as ratios relative to the ribosomal protein 36B4 (Rplp0) mRNA levels, and the qPCR primers used in this paper are listed in Supplementary Table S1.

### ChIP assay

ChIP assay was conducted following the protocol established by Upstate Biotechnology [4]. Briefly, chromatin lysates were prepared from C2C12 myotubes following crosslinking with 1% formaldehyde. Then, the lysates were precleared with protein A/G agarose beads (Yeasen) and immunoprecipitated using antibodies against Baf60c (PA5-41093, Invitrogen), Mef2c (ab211493, Abcam), or control IgG in the presence of bovine serum albumin (BSA) and salmon sperm DNA. The following day, each IP reaction was treated with protein A/G agarose beads for 2 h, after which the samples underwent extensive washing before reverse crosslinking. DNA was purified using a PCR Purification Kit (Qiagen) and subsequently analyzed by qPCR using primers located on the proximal *Musclin* promoter, *Dkk3* promoter, or *Gapdh* promoter (Supplementary Table S1).

### ATAC-seq analysis

An assay for ATAC-seq analysis was performed as described previously [4]. Briefly, 5 × 10^4^ nuclei from muscle tissues were used in each transposase reaction, followed by barcoding and library preparation. Finally, 150-bp paired-end reads used for DNA sequencing were trimmed to 38-bp paired-end reads by fastx_trimmer (https://anaconda.org/bioconda/fastx_toolkit) for further processing. The downstream analysis pipeline was adapted from a previous study [33]. Briefly, Macs2 (2.1.1.20160309) package and Deseq2 (v.1.20.0) package were used for broad peaks calling with the parameter of (--nomodel --shift -100 --extsize 200 -B --broad) and differential accessed peaks calling, respectively [16, 34]. Motif enrichment analysis and peak-associated gene annotation were performed by Hypergeometric Optimization of Motif EnRichment (HOMER) (V4.10) using peaks filtered by the corresponding criteria mentioned previously [35]. Browser tracks were visualized by IGV browser (V2.4.14) after normalizing the reads from each individual sample to its own library size [36]. The ATAC-seq data quality control analysis was performed using Ataqv (V1.0.0) package developed by Parker’s lab from the University of Michigan.

### Statistical analysis

Statistical analyses were carried out using GraphPad Prism 8. Statistical differences were evaluated using two-tailed unpaired Student’s *t*-test for comparisons between two groups or ANOVA and appropriate *post hoc* analyses for comparisons of multiple groups. For ITT and GTT, two-way ANOVA with multiple comparisons was used for statistical analysis. A *P* value of less than 0.05 (^*^P < 0.05; ^**^P < 0.01; ^***^P < 0.001) was considered statistically significant. No statistical method was used to predetermine the sample size. The experiments were not randomized and the investigators were not blinded to allocation during experiments and outcome assessment.

## Supplementary Material

loaf015_suppl_Supplementary_Materials

## Data Availability

The heatmap of representative secreted protein-encoding gene expression indicated in Fig. 1a was analyzed using the microarray data of skeletal muscles from BcMKO and control mice, which had been deposited in Mendeley data (https://doi.org/10.17632/28ks32hwxh.1). The heatmap of gene expression indicated in Fig. 2a was analyzed using the microarray data of C2C12-derived myotubes with or without *Baf60c* knockdown deposited in the Gene Expression Omnibus (GEO) datasets (GSE79925). Data associated with ATAC-seq of skeletal muscles from BcMKO and control mice presented in Fig. 3 were analyzed using the ATAC-seq datasets deposited in Mendeley data (https://data.mendeley.com/datasets/jxxxshvfsn/1). The bigwig files for H3K4me2 (GSE123879), H3K27ac (GSE123879), Mef2d (GSE43223), Six1 (GSE175999), Myf5 (GSE24852), and six4 (GSE66901) ChIP-seq data were downloaded from the GEO database. H3K4me3 (ENCSR000AHT) and MyoD1 (ENCSR000AIG and ENCSR000AIH) ChIP-seq data were downloaded from the Encyclopedia of DNA Elements (ENCODE) database. Any other data generated and/or analyzed during the current study are available from the corresponding authors on reasonable request.
