# Supplementary material for: Baf60c in skeletal muscle regulates adipose tissue thermogenesis via Musclin-mediated endocrine signaling
Source: Life Metab. 2025 May 4;4(4):loaf015. doi: 10.1093/lifemeta/loaf015 (PMC12201986; doi:10.1093/lifemeta/loaf015)
Supplement: loaf015_suppl_Supplementary_Materials [file loaf015_suppl_supplementary_materials.docx]

**Supplementary Data**


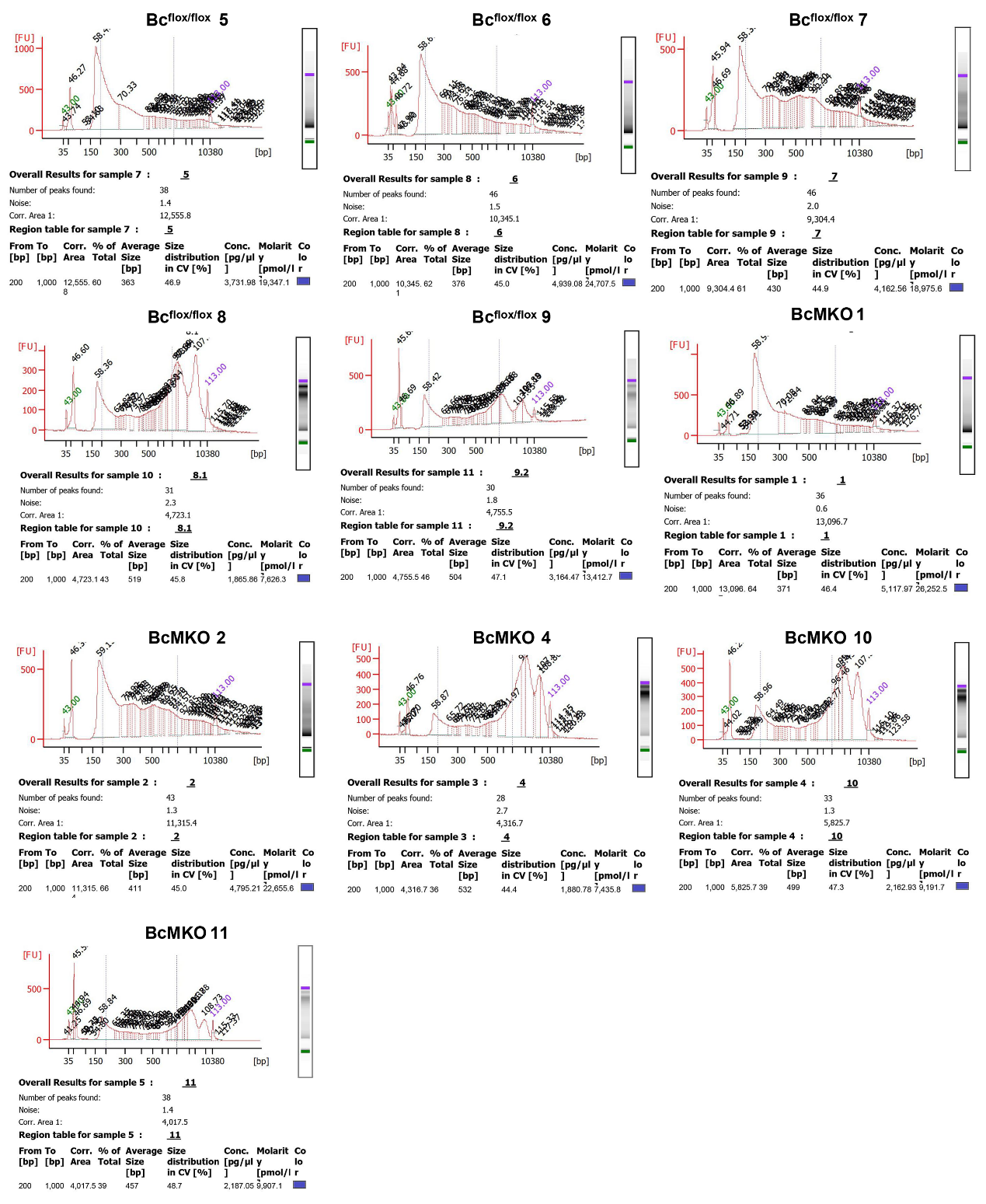


**Supplementary Figure S1** DNA fragment size distribution of the ATAC-seq samples.


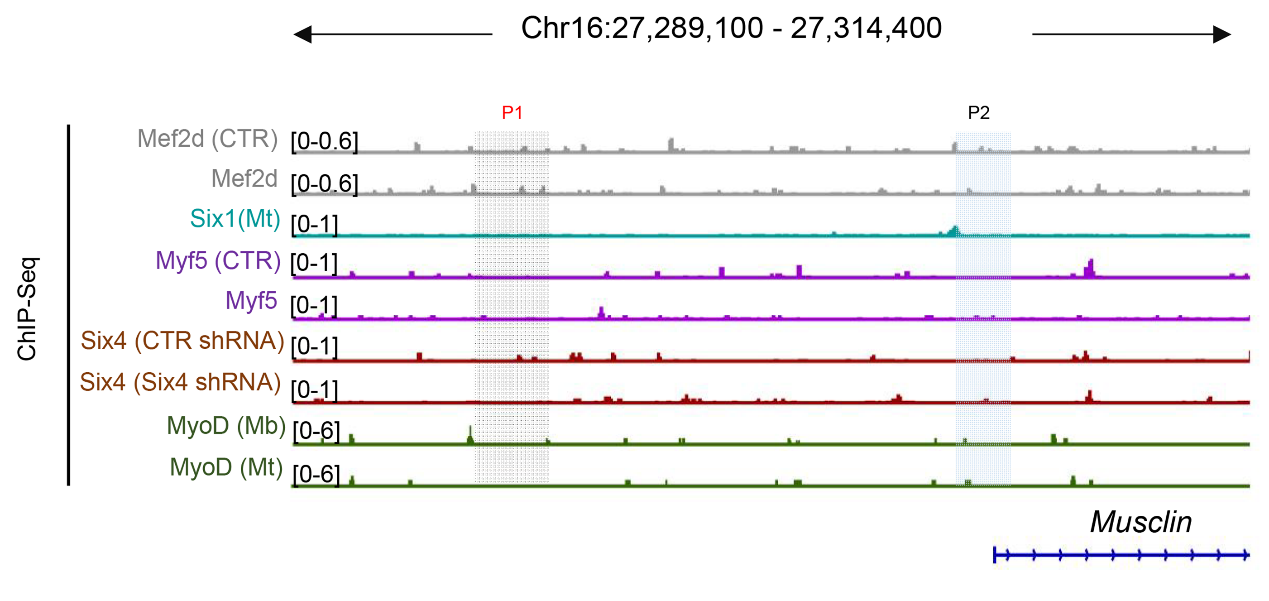


**Supplementary Figure S2** IGV views of indicated ChIP-seq data in the proximal region of the *Musclin* gene locus. The ChIP-seq data of Mef2d (GSE43223), Six1 (GSE175999), Myf5 (GSE24852), Six4 (GSE66901), and MyoD (ENCSR000AIG and ENCSR000AIH) are applied from the GEO and ENCODE datasets. Mef2d ChIP-seq analysis was performed on C2C12-derived myotubes that had been differentiated for 120 h. Mef2d (CTR) refers to the ChIP-seq result of nonspecific binding control to Mef2da2 (a muscle-specific isoform) antibody, performed using Mef2da2 antibody that was preincubated with peptides corresponding to the epitope of Mef2da2. Six1 ChIP-seq was performed in primary mouse myoblast-derived myotubes (48 h differentiation). Mt refers to myotube. ChIP-seq of Myf5 was alternated by chromatin tandem affinity purification sequencing (ChTAP-seq) in primary mouse myoblasts infected with retroviruses expressing Myf5-C-terminus TAP tag fusion protein (labelled as Myf5) or empty vector (labelled as Myf5 (CTR)). Six4 ChIP-seq analysis was performed on shNS C2C12 cell line (a control cell line) or shSix4 C2C12 (C2C12 with stable *Six4* knockdown using short hairpin RNA) after differentiation for 24 h. MyoD ChIP-seq analysis was performed in both myoblasts (Mb) and myotubes (Mt) with 60 h differentiation.


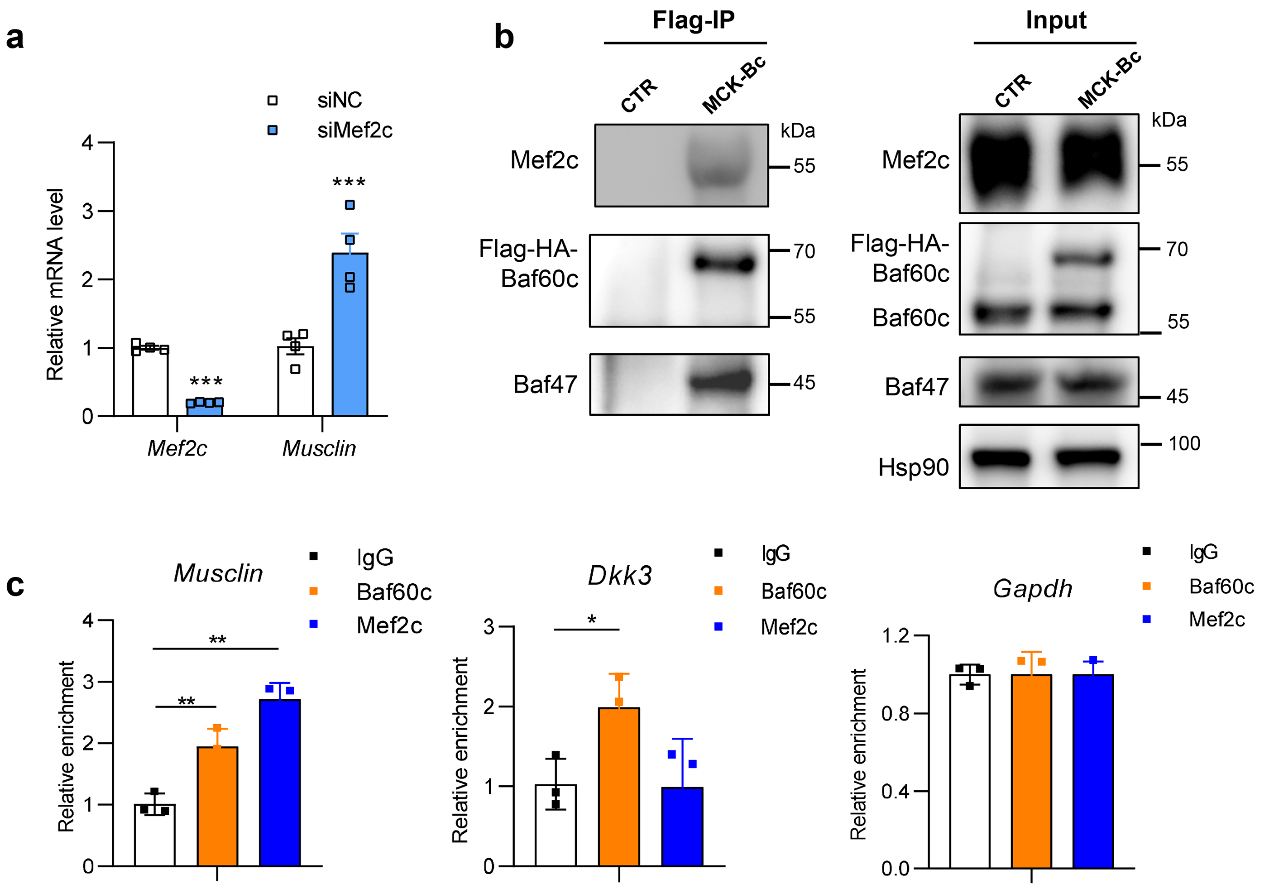


**Supplementary Figure S3** Baf60c physically interacts with Mef2c to regulate Musclin expression. (a) *Musclin* expression in *Mef2c* knockdown C2C12 myotubes. ^***^*P* < 0.001 by two-tailed unpaired Student’s *t*-test. (b) Physical interaction between Baf60c and Mef2c in the quadriceps using endogenous IP assay. IP, immunoprecipitation; Flag-HA-Baf60c, Flag-HA tagged Baf60c; WT, control mice; MCK-Bc, muscle-specific Flag-HA tagged *Baf60c* transgene mice. Baf47, another SWI/SNF complex component that interacts with Baf60c and works as a positive control in this Flag-IP assay. (c) Chromatin immunoprecipitation (ChIP) assay in C2C12 myotubes using antibodies against Baf60c, Mef2c, or control IgG (*n* = 3 technical replicates). ^*^*P* < 0.05; ^**^*P* < 0.01 by two-tailed unpaired Student’s *t*-test.


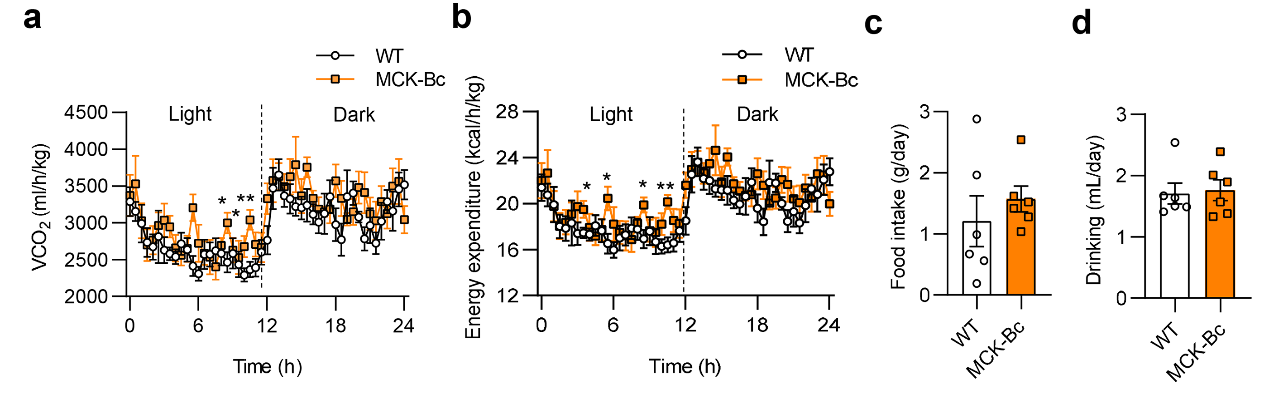


**Supplementary** **Figure. S4** Metabolic study in muscle-specific *Baf60c* overexpressed mice. (a−d) Phenotypes of MCK-Bc and control mice under high-fat diet feeding. The CO_2_ production rate (a), energy expenditure (b), food intake (c), and water consumption volume (d) were monitored at 30℃. ^*^*P* < 0.05; ^**^*P* < 0.01 by two-tailed unpaired Student’s *t*-test.


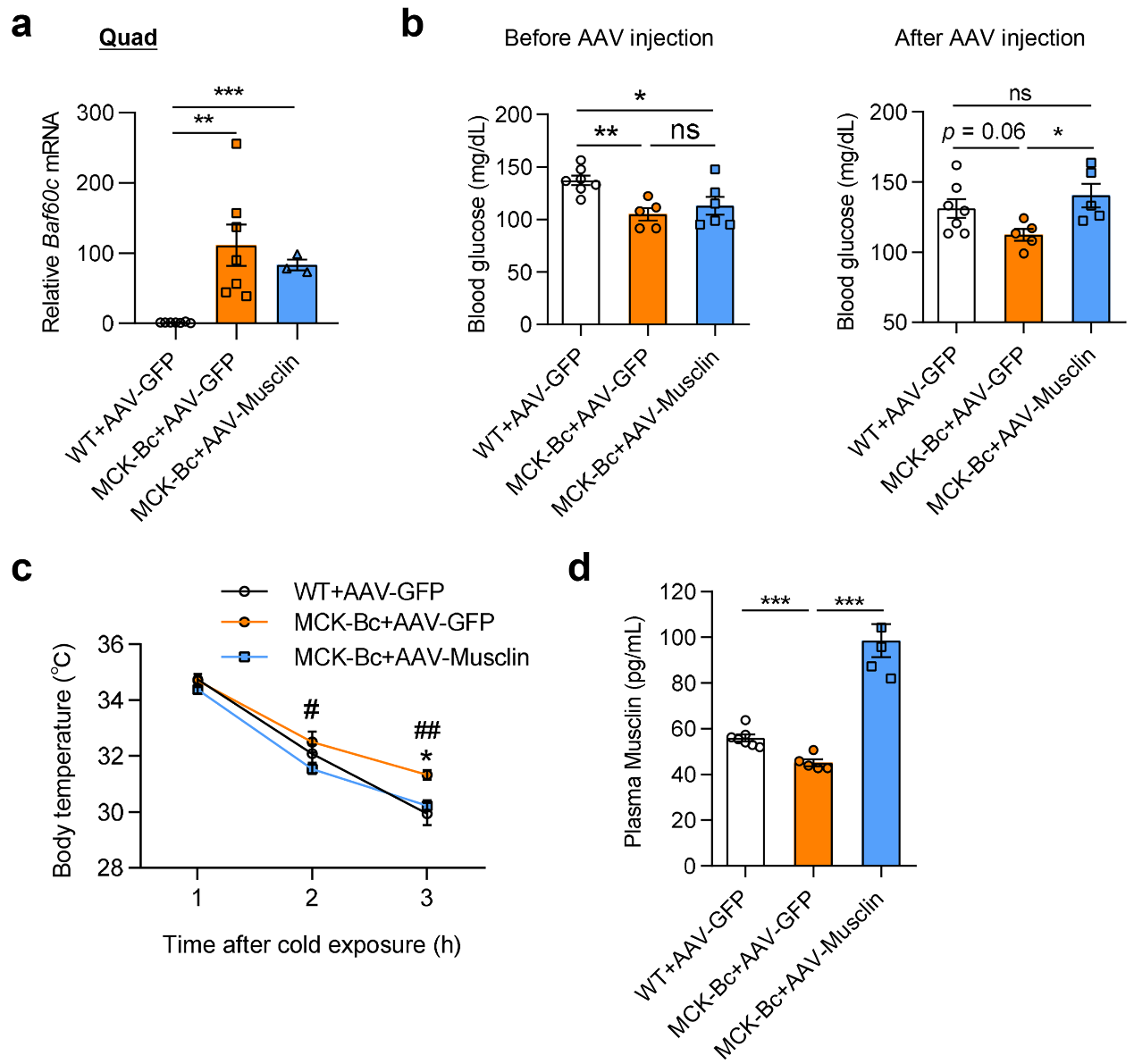


**Supplementary Figure S5** *Musclin* overexpression abolished muscle Baf60c-mediated metabolic beneficial effects. (a) Relative *Baf60c* mRNA levels in Quad muscles from *Baf60c*-overexpressed and control mice with either AAV-GFP or AAV-Musclin treatment (*n* = 5−7 per group). ^**^*P* < 0.01; ^***^*P* < 0.001 by two-tailed unpaired Student’s *t*-test. (b) Blood glucose levels before (*n* = 5−7 per group, left panel) and after (*n* = 5−7 per group, right panel) AAV injection in indicated mice. ^*^*p* < 0.05; ^**^*p* < 0.01; ns, no significance, by two-tailed unpaired Student’s *t*-test. (c) Core body temperature of indicated mice during acute cold (in a 4℃ chamber). ^*^*p* < 0.05, for WT + AAV-GFP versus MCK-Bc + AAV-GFP; ^#^*p* < 0.05; ^##^*p* < 0.01, for MCK-Bc + AAV-GFP versus MCK-Bc + AAV-Musclin, by two-way ANOVA with multiple comparisons. (d) Plasma Musclin level in indicated mice (*n* = 5−7 per group). ^***^*P* < 0.001by two-tailed unpaired Student’s *t*-test.

**Supplementary Table S1** Sequences of qPCR primers used in this study.

| *Gene name* | Forward primer (5’→3’) | Reverse primer (5’→3’) |
| --- | --- | --- |
| *Mouse* |  |  |
| *Rplp0* | GAAACTGCTGCCTCACATCCG | GCTGGCACAGTGACCTCACACG |
| *Musclin* | CTCAGCTGGGTCTGTAGAGCATAG | TACCAAACCGCTTTTTTGAATGAT |
| *Baf60c* | AGGCTTACATGGACCTCCTAG | CATCAGAGTCTTCCGCATCAG |
| *Mef2c* | GAGCGTGCTGTGCGACTGT | CGTGCGGCTCGTTGTACTC |
| *Ldhb* | GCACCATCATCGTGGTTTCC | GGTAGCGGAATCGAGCAGAA |
| *Cd36* | TTAGATGTGGAACCCATAACTGGA | TTGACCAATATGTTGACCTGCAG |
| *Cidea* | GCAGCCTGCAGGAACTTATCAGC | GATCATGAAATGCGTGTTGTCC |
| *Pgc1α* | AGCCGTGACCACTGACAACGAG | GCTGCATGGTTCTGAGTGCTAAG |
| *Pgc1β* | GCTCCAGGAGACTGAATCCAGAG | CTTGACTACTGTCTGTGAGGC |
| *Prdm16* | CGGAAGAGCGTGAGTACAAATG | TCCGTGAACACCTTGACACAGT |
| *Ucp1* | GGCATTCAGAGGCAAATCAGCT | CAATGAACACTGCCACACCTC |
| *Hsl* | GAGACACCAGCCAACGGATAC | TTTTGCGGTTAGAAGCCACAT |
| *Mgll* | CTCCACAGAATGTTCCCTACCA | ATCATAACGGCCACAGTGTTC |
| *Acat1* | ACCAGATGTGGTGGTGAAAGA | GGCAGCTGTTATTGTGCCATT |
| *Pdhb* | GAAAGGCAAGGGACCCACAT | GGTCTGATGGTGCGCAGATT |
| *Ldhb* | GCACCATCATCGTGGTTTCC | GGTAGCGGAATCGAGCAGAA |
| *Atgl* | GTCCTTCACCATCCGCTTGTT | GATGCTACCCGTCTGCTCTTT |
| *Lpl* | CAGCAAGACCTTCGTGGTGA | ATAATGTTGCTGGGCCCGAT |
| **ChIP qPCR primers** | | |
| Relative to TSS | Forward primer (5’→3’) | Reverse primer (5’→3’) |
| *Dkk3 promoter ~ −180* | TAACTCACAGTCCACGTTCC | AGGTTTTCTGTGACCCAGGA |
| *Musclin promoter ~ −12,000* | GGCGACTCTAACCTCTGCATT | ACTGTGCTGTGTCCCTGAAT |
| *Gapdh promoter* | CATCAAGAAGGTGGTGAAGCAGG | ACCAGGAAATGAGCTTGACAAAGTT |
| TSS, Transcriptional start site | | |
| **siRNA primers** |  |  |
|  | Forward primer (5’→3’) | Reverse primer (5’→3’) |
| *siMef2c* | CCCACCUGGCAGCAAGAACAC | GUUCUUGCUGCCAGGUGGGAU |
